# The Causal Relationship between Immune-Mediated Inflammatory Diseases and Aortic Aneurysm: A Bidirectional Two-Sample Mendelian Randomization Study

**DOI:** 10.1155/2024/2474118

**Published:** 2024-10-09

**Authors:** Sijia Sun, Jie Li, Mengxian Sun, Jie He, Songtao Tan, Ge Wang, Yuan Zheng, Xiaoping Fan

**Affiliations:** ^1^Second Clinical Medical College, Guangzhou University of Chinese Medicine, Guangzhou, China; ^2^Department of Cardiovascular Surgery, Guangdong Provincial Hospital of Chinese Medicine, The Second Affiliated Hospital of Guangzhou University of Chinese Medicine, Guangdong, China

## Abstract

**Methods:**

We sourced genetic association data from public genome-wide association study databases for populations of European ancestry. Adhering to MR principles, we identified valid instrumental variables from genetic variants. A range of statistical methods were applied for MR analysis, with the inverse variance weighted (IVW) method emerging as the most reliable estimator of causality in this context.

**Results:**

The causal estimates obtained using the IVW method revealed a significant association between genetically predicted AA and rheumatoid arthritis (RA; OR = 1.06, 95% CI = 1.01–1.12, *P*=0.029). Conversely, genetically predicted RA showed nonsignificant causal estimates of AA (OR = 0.97, 95% CI = 0.92–1.02, *P*=0.204). Additionally, there was no evidence to suggest that AA may increase the risk of inflammatory bowel disease (IBD), Crohn's disease (CD), ulcerative colitis (UC), systemic lupus erythematosus (SLE), and psoriasis (PSO). The sensitivity analysis confirmed the absence of heterogeneity or horizontal pleiotropy effects.

**Conclusion:**

Our findings shed light on the causal effects between genetically predisposed AA and RA. They also suggest the potential clinical utility of human leukocyte antigen (HLA) risk genetic markers for developing personalized treatment and prevention strategies.

## 1. Introduction

Immune-mediated inflammatory diseases (IMIDs) are a spectrum of disorders characterized by a variety of clinical manifestations, and they are on the rise in terms of prevalence and health impact [1]. While traditional risk factors such as smoking [2], metabolic syndrome [3], and obesity [4] have been linked to IMIDs, they do not account for the full extent of the increased cardiovascular risk observed in these conditions. Aortic aneurysms (AA), a serious and often silent cardiovascular condition, typically goes undetected until a critical rupture occurs, at which point, surgical repair may be the only option [5]. Epidemiological studies have suggested a connection between AA and an elevated risk of IMIDs, including rheumatoid arthritis (RA) [6], systemic lupus erythematosus (SLE) [7], and psoriasis (PSO) [8]. However, these findings are inconsistent [8, 9, 10] and may be confounded by factors such as concurrent medications, unknown comorbidities, changes in disease severity, and misclassification [6, 7, 8, 9, 10]. The relationship between inflammatory bowel disease (IBD) and the incidence of AA is particularly understudied, with only a few case reports suggesting a link between Crohn's disease (CD) [11, 12] and ulcerative colitis (UC) [13] in patients with aneurysm or arteritis. Despite some evidence of a correlation between IMIDs and AA, the precise causal relationship between these conditions remains elusive. This ambiguity poses challenges for clinical management and prevention strategies for both IMIDs and AA. To address this, we require more robust analytical methods.

Mendelian randomization (MR) is a powerful analytical tool used to infer causal relationships by leveraging genetic variants from genome-wide association studies (GWAS) [14] to serve as proxies for exposures and outcomes. Single nucleotide polymorphisms (SNPs), as genetic variants strongly associated with exposures, are employed as instrumental variables (IVs) to estimate their potential impact on disease outcomes [15]. Given that genetic mutations occur before birth and are thus not influenced by confounding factors or reverse causation, MR provides a means to estimate causality with reduced bias [16]. In this study, we harnessed publicly available datasets from large-scale GWAS of European ancestry to conduct a bidirectional two-sample MR analysis. Our goal is to elucidate the direction and strength of the causal associations between IMIDs and AA and to deepen our genetic understanding of the interplay between these complex diseases.

## 2. Methods

### 2.1. Data Sources and Study Design

**Figure 1:**
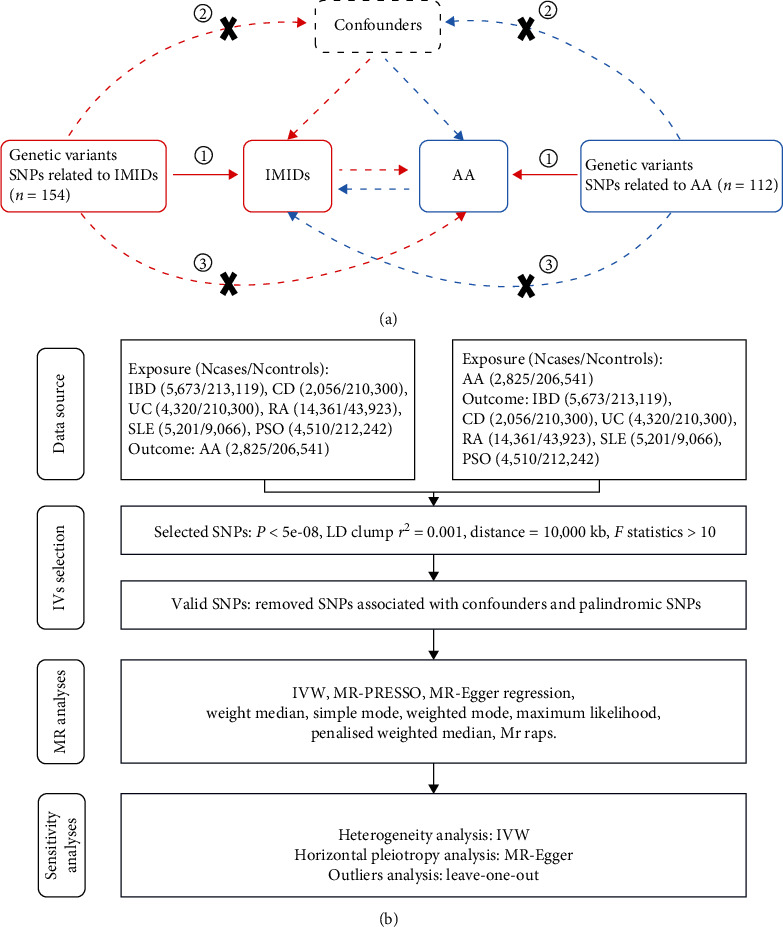
(a) Schematic view of this MR study for the association between IMIDs and AA. ①Relevance: the genetic variant must be robustly associated with the exposure of interest. ②Independence: the genetic variant must be independent of any confounders that affect the exposure. ③Exclusion restriction: the genetic variant affects the outcome only through its effect on the exposure and not through any alternative pathways. IMIDs, Immune-mediated inflammatory diseases, including inflammatory bowel disease (IBD), Crohn's disease (CD), ulcerative colitis (UC), rheumatoid arthritis (RA), systemic lupus erythematosus (SLE), and psoriasis (PSO), and AA, aortic aneurysm. (b) Detailed flowchart of the study design. IVs, instrumental variables.

We conducted a bidirectional two-sample MR study using a variety of publicly accessible GWAS datasets. A schematic overview of our study design is depicted in Figure 1(a). Our investigation encompassed IMIDs, including RA, PSO, SLE, and IBDs such as CD and UC. While both CD and UC are characterized by bowel mucosa inflammation, they each are present with unique clinical and pathological profiles. This prompted a detailed comparative analysis of these two IBD subtypes. We accessed genetic association data for IMIDs and AA through the IEU OpenGWAS database project, available at IEU OpenGWAS Database (https://gwas.mrcieu.ac.uk/). This resource offers a comprehensive repository of GWAS outcomes, standardizing raw data into a uniform format accessible via MR-Base, facilitating MR analysis [17]. In the selection of datasets for exposures and outcomes, we prioritized those with significant sample sizes and recent updates, primarily drawing from the FinnGen biobank and the European Bioinformatics Institute. For our analysis, we utilized specific GWAS IDs: “finn-b-K11_IBD”, “finn-b-K11_CROHN”, and “finn-b-K11_ULCER” for IBD, CD, and UC, respectively; “finn-b-L12_PSORIASIS” for PSO; “ebi-a-GCST90013534” [18] for RA; “ebi-a-GCST003156” [19] for SLE; and “finn-b-I9_AORTANEUR” for AA. All datasets were derived from individuals of European ancestry. *Supplementary table 1* provides further details on these GWAS datasets. In line with the STROBE-MR guidelines [20], we present the study's design, methodologies, and a thorough analysis of the results, ensuring transparency and reproducibility in our research findings.

### 2.2. Genetic Instrumental Variables Selection

A stringent genome-wide significance threshold of *P* < 5e-08 was set to identify SNPs strongly associated with IMIDs. In the initial search for proxy SNPs for AA, this threshold yielded only two potential IVs. To facilitate the identification of additional SNPs, we relaxed the threshold to a more lenient value (*P* < 5e-06). To avoid double-counting the contributions of SNPs to the phenotype of interest, we excluded linked variants by applying linkage disequilibrium correlation parameters, setting the threshold for *r*^2^ at 0.001 and the distance at 10,000 kb [21]. This strategy ensures the inclusion of only independent genetic variants in our analysis, thereby minimizing bias. Furthermore, the pleiotropic effects of SNPs can introduce biases, leading us to preliminarily control for the pleiotropy of genetic variants during the selection of IVs. For this purpose, we used the online tool PhenoScanner v2 to eliminate SNPs associated with traits of confounding and outcome data as reported in existing literature [22]. After correlation filtering, removal of linkage disequilibrium, and exclusion of potentially confounding IVs, all data retained at least three SNPs associated with traits of IMIDs and AA. Finally, we evaluated the strength of the IVs by calculating the genetic instruments *R*^2^ and *F* statistic. The formulas for *R*^2^ and *F* statistic are as follows: *R*^2^ = (2*β*^2^ × MAF × (1−MAF))/(2*β*^2^ × MAF × (1−MAF) + 2*β*^2^ × MAF × (1−MAF) × N × SE), and F = *R*^2^ × (N−k−1)/(k × (1−*R*^2^)) (where *β* represents the effect estimate of the SNP in the exposure, MAF is the minor allele frequency, N is the sample size of the GWAS datasets, k is the number of IVs, and SE is the standard error of the genetic effect) [23].

### 2.3. Statistical Analysis

This MR analysis was performed using the “TwoSampleMR”, “MR-PRESSO”, and “mr. raps” packages in R software (version 4.2.2). A variety of MR methods, including inverse variance weighted (IVW) with both fixed and random effects models [24], MR-Egger regression [25], weighted median [26], penalized weighted median [27], simple mode [28], weighted mode [28], and maximum likelihood [29], were utilized to estimate the association between the selected IVs and each outcome. In our study, the primary estimated effects were derived from the IVW method under fixed effects. The IVW method yields unbiased estimates when the chosen SNPs are valid IVs [30]. Other methods were used as supplementary approaches. These methods ensured robust results in terms of both efficiency and strength [31]. In the heterogeneity analysis, we employed a modified Cochran's Q test and MR-Egger regression to assess data quality and identify potential errors in harmonizing genetic variants. Consistent results across these two tests, with a *P* value greater than 0.05, indicated successful harmonization and absence of significant heterogeneity [32]. In the horizontal pleiotropy analysis, the MR-PRESSO approach was used to identify and remove outliers, with a global test *P* value greater than 0.05 suggesting no existence of horizontal pleiotropy [33]. Additionally, we applied MR-Egger regression to detect potential pleiotropic bias [25]. To further validate the reliability of the MR results, we conducted a leave-one-out analysis, where we recalculated the MR estimates after sequentially removing each SNP. Finally, we visually presented the main results of the MR analysis using scatter plots, forest plots, and funnel plots.  Figure 1(b) provides a detailed flowchart of our MR study.

## 3. Results

### 3.1. The Causal Effect of IMIDs on AA

**Figure 2:**
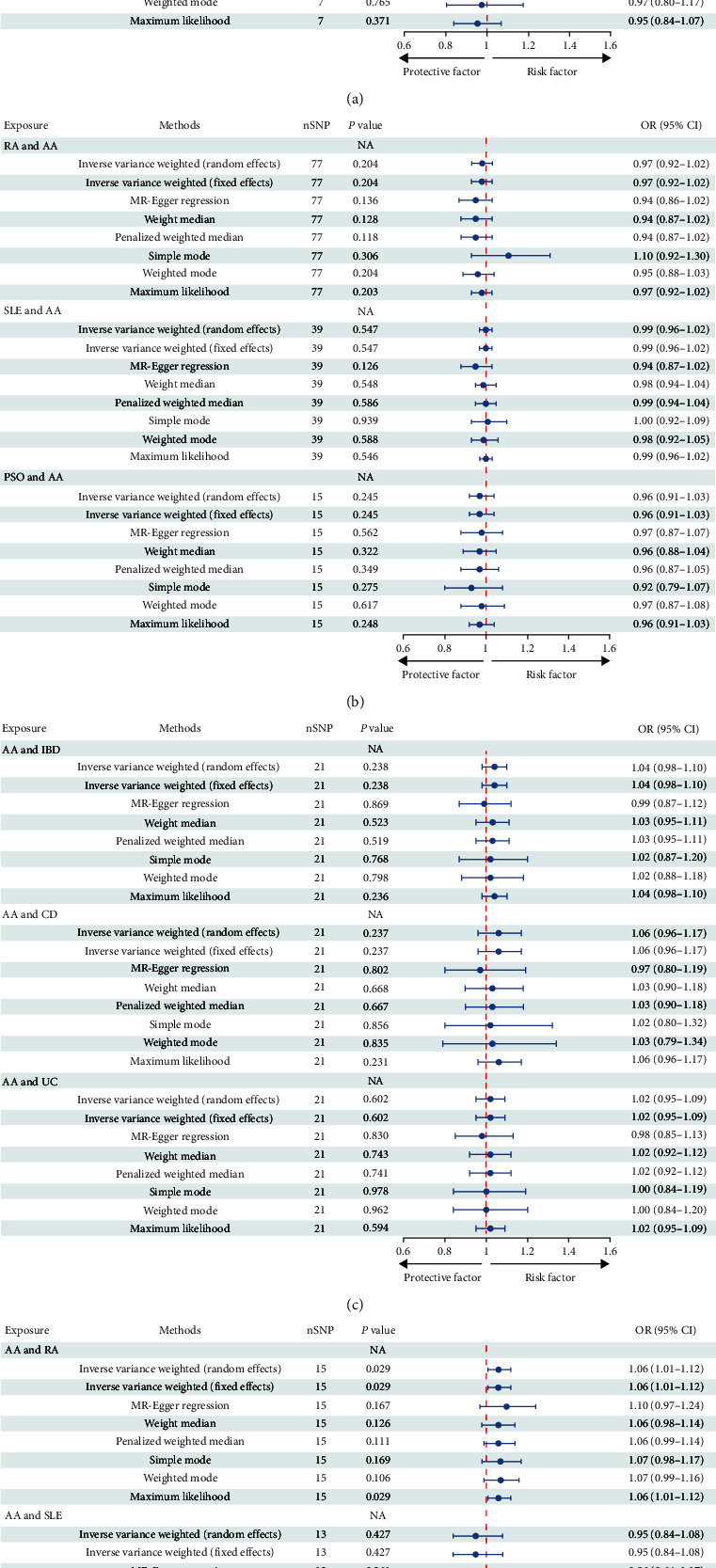
Main result of estimates from MR analysis with forward and reverse direction. (a and b) Forward analysis. (c and d) Reverse analysis.

A total of 179 SNPs linked to IMIDs were identified, adhering to the established criteria for MR assumptions. We accounted for genetic confounders such as smoking, alcohol consumption, hypertension, dyslipidemia, obesity, and glucocorticoid use [5, 34, 35]. After the exclusion of palindromic SNPs and those correlated with these confounders, 154 independent SNPs were retained. These SNPs were categorized as follows: 10 SNPs associated with IBD and AA, 6 with CD and AA, 7 with UC and AA, 77 with RA and AA, 39 with SLE and AA, and 15 with PSO and AA. The *F* statistics for these IVs were computed, with detailed characteristics provided in *Supplementary table 2*. The MR analysis, utilizing the IVW model with fixed effects, did not reveal a causal link between IMIDs and AA. Specifically, ORs and 95% CIs for IBD and AA were 0.91 (0.81–1.01), *P*=0.072; for CD and AA, 0.97 (0.88–1.07), *P*=0.544; for UC and AA, 0.95 (0.84–1.07), *P*=0.363; for RA and AA, 0.97 (0.92–1.02), *P*=0.204; for SLE and AA, 0.99 (0.96–1.02), *P*=0.547; and for PSO and AA, 0.96 (0.91–1.03), *P*=0.245. The consistency of these results with other MR methods is illustrated in Figure 2. Sensitivity analyses, including the Cochran's Q test, indicated no significant heterogeneity (all *P* values > 0.05; Table 1). Furthermore, the intercept of the MR-Egger regression did not deviate from zero, suggesting the absence of horizontal pleiotropy (Table 1). This was supported by the symmetrical distribution in the funnel plot (*Supplementary figure 1*). Additionally, the leave-one-out analysis confirmed the stability of the overall odds ratio, with no single SNP exerting a significant influence (*Supplementary figure 2*). Collectively, these findings suggest that there is no causal association between the examined IMIDs and AA.

### 3.2. The Causal Effect of AA on IMIDs

The initial screening process yielded 138 potentially independent SNPs that fulfilled the inclusion criteria. We excluded SNPs associated with recognized confounders—such as depression, anxiety, smoking, glucocorticoid use, nonsteroidal anti-inflammatory drug consumption, obesity, or malnutrition—that have established causal relationships with IMIDs [36, 37, 38, 39]. Following harmonization of the exposure and outcome datasets, 112 SNPs were retained for analysis. This selection comprised 21 SNPs each for the relationship between AA and IBD, CD, and UC; 15 SNPs for AA and RA; 13 SNPs for AA and SLE; and 21 SNPs for AA and PSO (*Supplementary table 3*). These 112 independent SNPs served as IVs to investigate the influence of AA on IMIDs. The IVW method indicated that individuals predisposed to AA have a 6% increased risk of developing RA (OR = 1.06, 95% CI = 1.01–1.12, *P*=0.029), as illustrated in Figure 2 and *Supplementary figure 3*. This causal association was further supported by the MR-PRESSO method (OR = 1.06, 95% CI = 1.01–1.11, *P*=0.019). Cochran's Q test did not reveal heterogeneity among the IVs related to RA (Q = 11.82, *P*=0.621). Additionally, both MR-PRESSO and MR-Egger regression analyses provided no indication of horizontal pleiotropy, with the latter showing an intercept of −0.01 (*P*=0.570; Figure 2). However, the genetically predicted effects of AA on IBD, CD, UC, SLE, and PSO did not achieve statistical significance in either the IVW or MR-PRESSO methods, as detailed in Figure 2 and Table 2. Sensitivity analyses, encompassing Cochran's Q test and Egger intercept, confirmed the absence of underlying heterogeneity and horizontal pleiotropy across the examined associations (all *P* values > 0.05; Table 1).

## 4. Discussion

To our knowledge, this is the inaugural MR study investigating the relationship between exposure to IMIDs and the incidence of AA. Utilizing a genetic approach, our research highlighted that an elevated genetically predicted risk of AA was significantly correlated with the onset of RA. Conversely, no substantial causal estimate was detected from RA to AA. Moreover, our MR findings suggested that other IMIDs, such as PSO, SLE, IBD, CD, and UC, do not have a genetic causal relationship with AA. Our discoveries regarding the new associations between AA and RA underscore the necessity for early screening for potential copathogenic genes of RA in AA patients.

AA is characterized as a chronic inflammatory process of the aorta, where the activation of inflammatory proteases and the remodeling of the extracellular matrix are pivotal in disease progression [40]. Immune cells are a primary source of secreted inflammatory factors and proteases [41]. Inflammatory infiltration are frequently observed in the aortic adventitia of patients with inflammatory rheumatic diseases. Pathological findings have shown increased expression of TNF-*α*, IL-18, and IL-33 in the aortic adventitia of RA patients, suggesting an expedited development of vascular inflammation [42]. Studies on the mechanisms of inflammatory pathways have identified commonalities in key immune cell populations and the release of shared inflammatory mediators in PSO and cardiovascular diseases [43]. Furthermore, animal experiments have shown that IL-17 and IL-23, crucial inflammatory cytokines in PSO, regulate the phenotype switching of vascular smooth muscle cells and the release of pro-inflammatory cytokines [44, 45]. Bioinformatics and immune cell infiltration analysis have revealed that CXCL1-mediated neutrophil activation is a shared immune-inflammatory regulatory mechanism in abdominal aortic aneurysm (AAA) and SLE [46]. Additionally, IBD, when accompanied by gut microbiota dysbiosis, can stimulate neutrophil extracellular trap formation and weaken the aortic wall [47]. These findings clarify that IMIDs are independent risk factors for AA and may induce vascular damage through the recruitment and activation of immune cells.

Over the past two decades, numerous epidemiological studies have hinted at a potential connection between IMIDs and AA. For instance, research by Khalid et al. disclosed a correlation between PSO and an elevated risk of abdominal AA. This risk intensifies with the severity of PSO, even when considering established cardiovascular risk factors [8]. A cohort study by Chiu et al. [9] arrived at a similar conclusion. However, another study found no significant link between the severity of PSO and AA, suggesting that surveillance bias could potentially lead to an inverse conclusion [10]. Several analyses may provide plausible explanations for these differing results. On the one hand, patients with PSO are more likely to undergo more diagnostic and medical examinations compared to the control group, which could result in an underestimation of the diagnosis rate of AA in mild cases. On the other hand, the history of corticosteroid medication use is also a significant confounder. It has been reported that even low-dose intake of corticosteroids could hasten the development of cardiovascular diseases [34, 36, 48]. Our MR findings did not identify a significant genetic association between PSO and AA. Similarly, no direct genetic causal relationships were found between SLE, IBD, CD, and UC with AA. These results could be attributed to the elimination of more confounding factors when determining genetic instrumental variables in our study. SNPs associated with phenotypes of risk factors for AA, such as obesity, dyslipidemia, hypertension, alcohol consumption, smoking, and corticosteroid use, were excluded from our analysis [5, 34, 35]. More than half of SLE patients reportedly have multiple complications, with hypertension being the primary cause of cardiovascular complications in SLE [49]. Patients with active SLE are subject to hypertension not only due to secondary renal impairment but also due to a multifaceted array of factors independent of lupus nephritis [49, 50]. These include systemic vascular endothelial cell dysfunction, elevated levels of endothelin-1 [51], activation of the renin–angiotensin–aldosterone system (RAAS) [52], and a reduction in efferent vagal nerve tone [53]. Each of these factors contributes independently to the overall vascular stress and contraction observed in individuals with active SLE, thus increasing the risk of developing aortic dilatation. Smoking and obesity have been identified as environmental and lifestyle risk factors for IBD, which may independently contribute to the development and progression of aneurysms by altering the gut microbiome and influencing dietary habits, respectively [54, 55]. However, excluding genetic variants related to confounding factors may also omit some that truly influence the onset of IMIDs, particularly those variants that are simultaneously associated with confounding factors. Additionally, combining the analysis of thoracic aortic aneurysm (TAA) with AAA might have diminished the chances of identifying a significant association. AAs are a group of diseases characterized by segmental heterogeneity, with notable differences in the morphology and structural composition between the thoracic and abdominal segments of the aortic wall [56]. The thoracic aorta, being closer to the heart, withstands higher pressures from cardiac output and contains a higher proportion of elastin in its media. In contrast, the adventitia of the abdominal aorta is thicker than that of the thoracic segment, and the ratio of elastin to collagen is reduced [57]. The adventitia is primarily composed of connective tissue and is rich in blood supply, which can be particularly sensitive to inflammatory mediators and immune reactions. Consequently, causative effect of genetic correlation between certain IMIDs and AAA may be obscured. Research by Shovman et al. [6] explored the relationship between RA and AA, revealing a higher incidence of AA in patients with RA. Ntusi et al. [58] also reported that RA patients, even without cardiovascular risk factors, exhibited a similar degree of vascular dysfunction as patients with cardiovascular risk factors. While epidemiological studies provide causal support for the role of immune-mediated inflammatory processes in the etiology of AA, the direction of causality may be compromised by confounding biases and measurement errors, potentially leading to contradictory results. These association studies prompted us to infer causal relationships between RA and AA. However, our MR results show that the causal estimate of AA for RA is significant. This finding supports the novel associations between AA and RA.

The precise mechanism through which a genetic predisposition to AA heightens the risk of RA is not fully understood. Nonetheless, existing literature proposes a hypothesis for this potential causal relationship. The shared epitope found in human leukocyte antigen (HLA) molecules might act as copathogenic genetic risk factors in the development of both AA and RA [59]. While AAA frequently coexist with atherosclerotic changes, mounting evidence suggests that AAA is a complex T cell autoimmune disease, driven by specific antigens, with genetics playing a significant role in its pathogenesis [59, 60]. PCR amplification and sequencing analyses have verified the presence of identical *α*-chain and *β*-chain T cell receptor clone transcripts within the same region of T cell populations in AAA lesions [60, 61]. The amino acid residue DR*β*Gln70, found in over 80% of AAA lesion tissues, is a component of a sequence motif within the antigen-binding groove of the HLA-DRB1 molecule, known as the shared epitope (SE) [60, 62]. Molecules of HLA-DRB1 carrying this shared epitope increase the predisposition to RA and other autoimmune diseases [37, 63]. Among the numerous genetic risk loci associated with RA, HLA-DRB1 is often referred to as the locus with the strongest risk effect. Studies have shown that RA patients who test positive for anticitrullinated peptide antibodies often exhibit more severe erosive arthritis and carry HLA molecules with SE features. Analysis of structural biology suggests that citrullinated antigens may bind more strongly to the peptide-binding groove [64, 65]. Furthermore, smoking significantly increases serum levels of anticyclic citrullinated peptide (anti-CCP) antibodies and is a prominent risk factor for RA patients with HLA-DRB1 SE. In the subgroup of RA patients who are RF+/anti-CCP2+, smoking and the presence of the HLA–DRB1 SE allele independently increase the risk of RA, and there is a significant interaction between these factors with an attributable proportion of 0.4. In the RF+/anti-CCP2− subgroup, although smoking does increase the risk of the disease, this risk is almost unaffected by the presence of the HLA-DRB1 SE allele. This indicates that smoking plays a significant role in RA patients who are positive for anti-CCP and carry the HLA-DRB1 SE genetic background [66]. As tobacco is also a major risk factor for AA [67], the gene–environment interaction between tobacco and SE may partially account for the frequent comorbidity of RA and AA observed clinically. Inflammation in AAA patients involves an *in situ* immune response at the aortic wall, suggesting that shared motifs of self-antigens identified in aortic tissue could be targets for autoimmunity recognition in the joints. In the pathogenesis of RA, collagen, particularly type II collagen, is recognized as an autoantigen that can trigger RA autoimmunity [68]. Current research has identified several putative self-antigens in AAA, including elastin and its fragments, collagen type I and III, and human microbial-associated glycoprotein-36 (MAGP-36) [59]. However, it remains unclear whether the epitopes of these putative self-antigens in AAA tissue are shared with the collagen autoantigens associated with RA. Information about the role of the HLA-DR gene in the pathogenesis of TAA is limited. A recent MR study investigating the causal impact of 731 immune cell types on TAA found that CD45+ HLA-DR + CD8+ T cells are a protective factor for TAA. This suggests that the role of HLA-DR labeled CD8+ T cells may differ between the thoracic and abdominal aorta [69]. Further studies are needed to investigate the association of TAA with HLA.

The limitations of our study merit careful consideration. First, while we have identified a correlation between genetic susceptibility to AA and an increased risk of RA, it is crucial to note that this finding should not be directly applied to predict the impact of interventions on RA risk in genetically susceptible individuals. Second, our research faced challenges in disentangling the causality between thoracic and abdominal AAs and IMIDs, which may have reduced our ability to detect significant associations. Third, the extensive polymorphism of HLA-DRB1 genes and the variability in allele distribution across ethnic groups are well-documented [70]. Given the heterogeneity of AA, the applicability of our findings in populations of European descent to other ethnic groups is uncertain. Future research should aim to include a more diverse demographic to validate and compare these findings across different ethnic groups. Last, our MR study did not consider potential nonlinear effects, which is a complex issue given the intricate pathways from genotype to phenotype. Further research is necessary to delve into these complexities and their implications for the future.

## 5. Conclusion

In conclusion, our study suggests a potential increase in the risk of RA associated with genetically predicted AA. However, we found no evidence to suggest that RA elevates the risk of AA. Furthermore, we did not identify any association between AA and other conditions such as IBD, CD, UC, SLE, and PSO. Our study underscores the existence of a potential genetic and causal nexus between genetic susceptibility to AA and the development of RA. This insight could pave the way for the utilization of HLA risk genetic markers in clinical practice, facilitating the advancement of tailored treatment and preventive strategies for RA.

## Figures and Tables

**Table 1:** Heterogeneity and pleiotropy analysis.

Exposure	Outcome	Heterogeneity			Pleiotropy	
		Methods	Cochran's Q	*P* value	Egger intercept (95% CI)	*P* value
IBD	AA	IVW	2.88	0.969	−0.02 (−0.06, 0.03)	0.491
CD	AA	IVW	3.28	0.656	0.00 (−0.06, 0.06)	0.929
UC	AA	IVW	2.48	0.871	0.00 (−0.05, 0.06)	0.911
RA	AA	IVW	72.14	0.604	0.01 (−0.01, 0.02)	0.353
SLE	AA	IVW	30.16	0.810	0.02 (−0.01, 0.05)	0.157
PSO	AA	IVW	11.51	0.645	0.00 (−0.04, 0.03)	0.897
AA	IBD	IVW	12.72	0.889	0.01 (−0.02, 0.05)	0.405
AA	CD	IVW	16.40	0.691	0.03 (−0.03, 0.08)	0.353
AA	UC	IVW	13.22	0.868	0.01 (−0.03, 0.05)	0.596
AA	RA	IVW	11.82	0.621	−0.01 (−0.04, 0.02)	0.570
AA	SLE	IVW	7.44	0.827	0.03 (−0.05, 0.10)	0.507
AA	PSO	IVW	18.99	0.522	0.01 (−0.03, 0.04)	0.772

**Table 2:** Results of supplemented MR methods.

Exposure	Outcome	MR			
		Methods	*β*	OR (95% CI)	*P* value
IBD	AA	MR-PRESSO	−0.06	0.94 (0.88, 1.00)	0.100
		Mr raps	−0.06	0.94 (0.83, 1.06)	0.320
CD	AA	MR-PRESSO	−0.03	0.97 (0.90, 1.05)	0.488
		Mr raps ^*∗*^	NA	NA	NA
UC	AA	MR-PRESSO	−0.06	0.94 (0.88, 1.01)	0.139
		Mr raps	−0.06	0.94 (0.83, 1.06)	0.336
RA	AA	MR-PRESSO	−0.04	0.96 (0.92, 1.01)	0.146
		Mr raps	−0.04	0.96 (0.91, 1.01)	0.117
SLE	AA	MR-PRESSO	−0.01	0.99 (0.96, 1.02)	0.593
		Mr raps	−0.01	0.99 (0.96, 1.03)	0.691
PSO	AA	MR-PRESSO	−0.01	0.99 (0.94, 1.04)	0.678
		Mr raps	0.00	1.00 (0.94, 1.05)	0.870
AA	IBD	MR-PRESSO	0.04	1.04 (0.99, 1.09)	0.154
		Mr raps	0.04	1.04 (0.97, 1.11)	0.273
AA	CD	MR-PRESSO	0.06	1.06 (0.97, 1.16)	0.207
		Mr raps	0.06	1.06 (0.95, 1.18)	0.290
AA	UC	MR-PRESSO	0.02	1.02 (0.96, 1.08)	0.529
		Mr raps	0.02	1.02 (0.95, 1.10)	0.613
AA	RA	MR-PRESSO	0.06	1.06 (1.01, 1.11)	0.019
		Mr raps	0.06	1.06 (1.00, 1.12)	0.034
AA	SLE	MR-PRESSO	−0.03	0.97 (0.88, 1.07)	0.561
		Mr raps	−0.04	0.96 (0.87, 1.07)	0.453
AA	PSO	MR-PRESSO	0.01	1.01 (0.95, 1.08)	0.691
		Mr raps	0.01	1.01 (0.94, 1.09)	0.791

^*∗*^For method of Mr raps, it requires a minimum of seven SNPs.

## Data Availability

The summary genetic association datasets for examined IMIDs and AA are accessible through the IEU OpenGWAS Database Project (https://gwas.mrcieu.ac.uk/) or the published article and its supplementary files. For further inquiries, please contact the corresponding author.

## Supplementary Materials

Supplementary 1 Table 1: characteristics of GWAS datasets in the study.

Supplementary 2 Table 2: instrumental variables related to immune-mediated inflammatory diseases.

Supplementary 3 Figure 1: funnel plot for IVW and MR-Egger method.

Supplementary 4 Figure 2: leave-one-out sensitivity analysis.

Supplementary 5 Table 3: instrumental variables related to aortic aneurysm.

Supplementary 6 Figure 3: scatter plot with all IVs.
