# Supplementary figures and images for: The Causal Relationship between Immune-Mediated Inflammatory Diseases and Aortic Aneurysm: A Bidirectional Two-Sample Mendelian Randomization Study
Source: J Immunol Res. 2024 Oct 9;2024:2474118. doi: 10.1155/2024/2474118 (PMC11483648; doi:10.1155/2024/2474118)

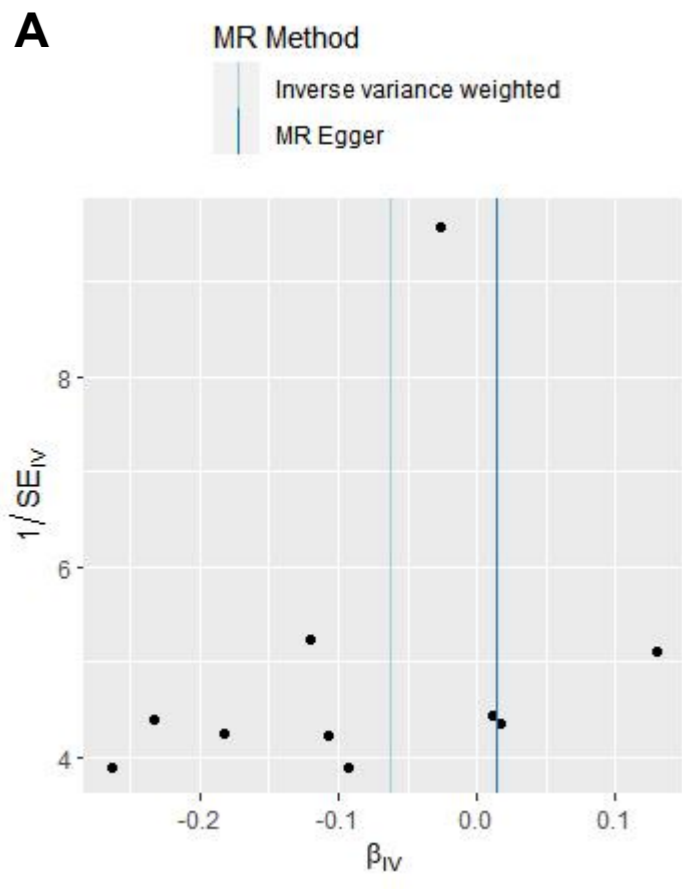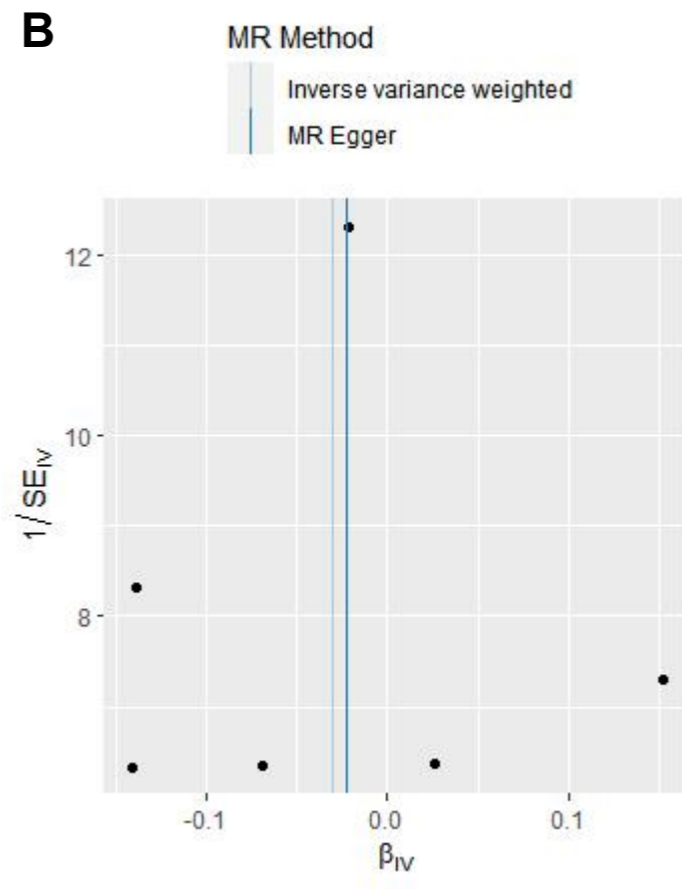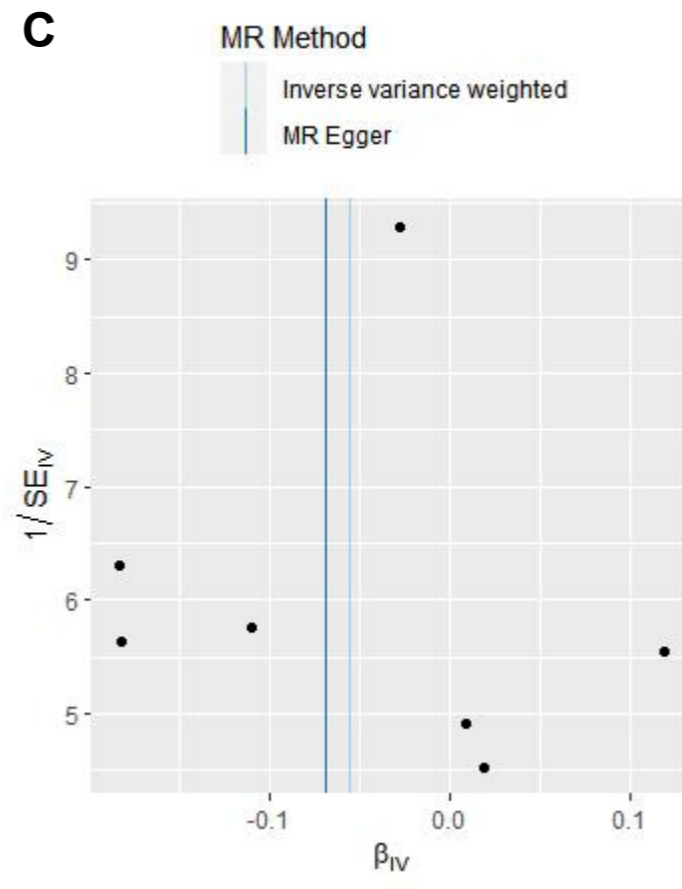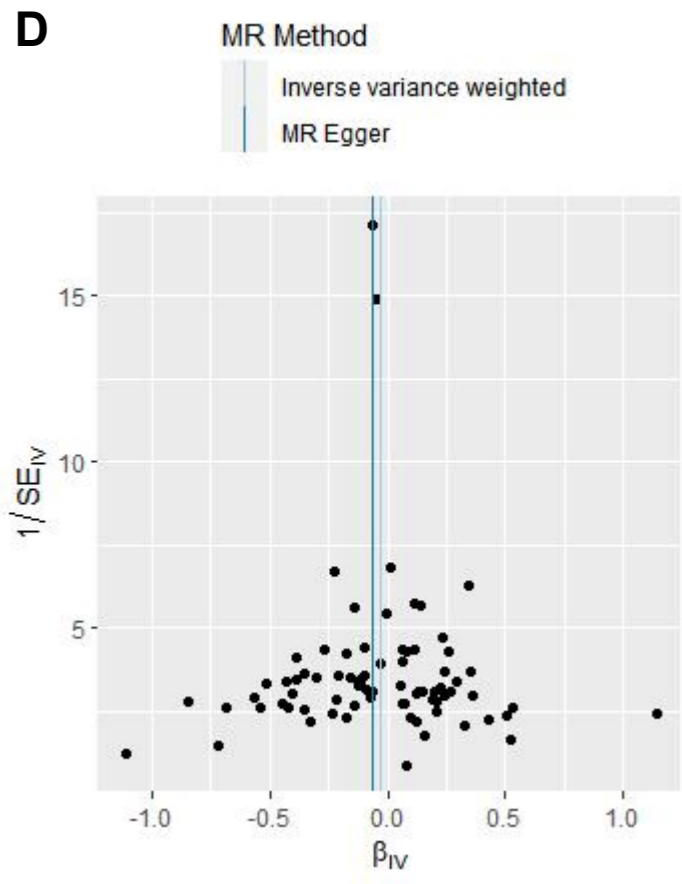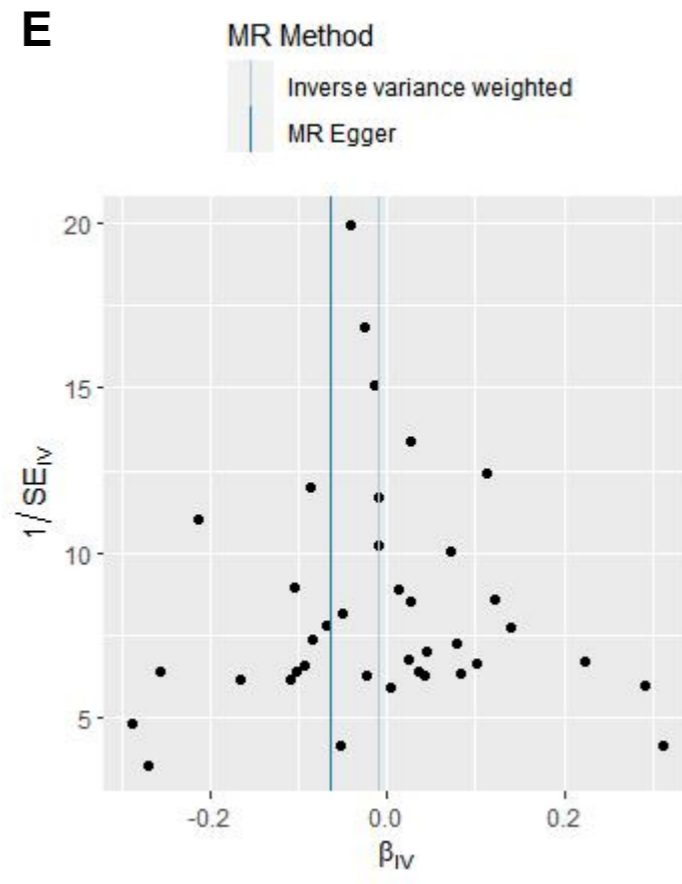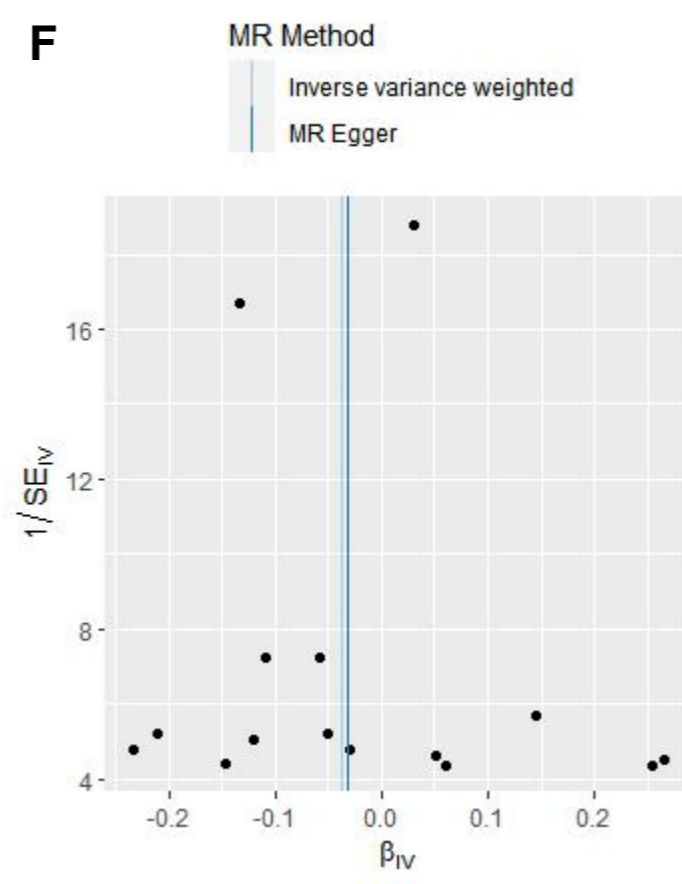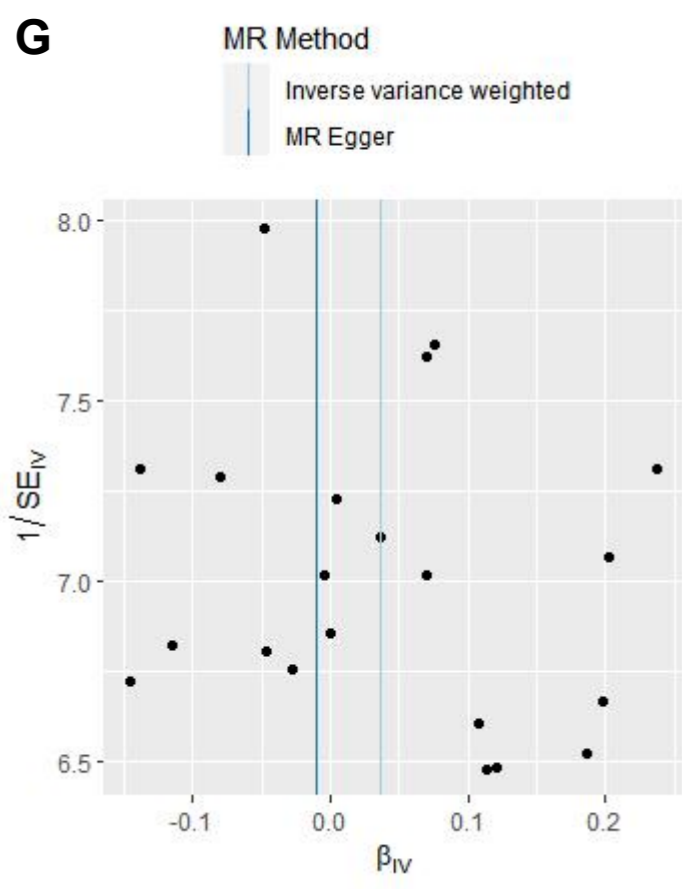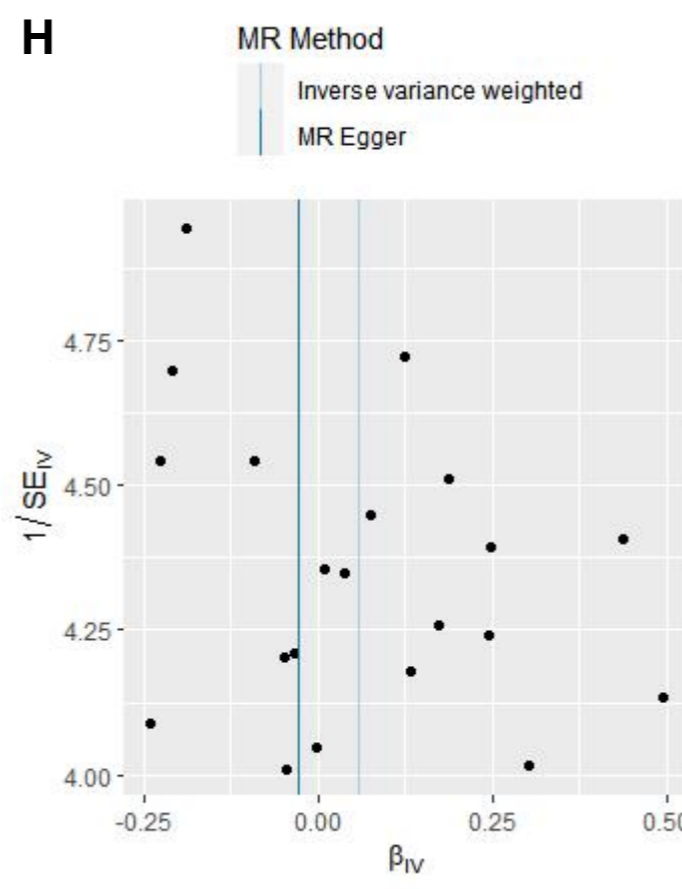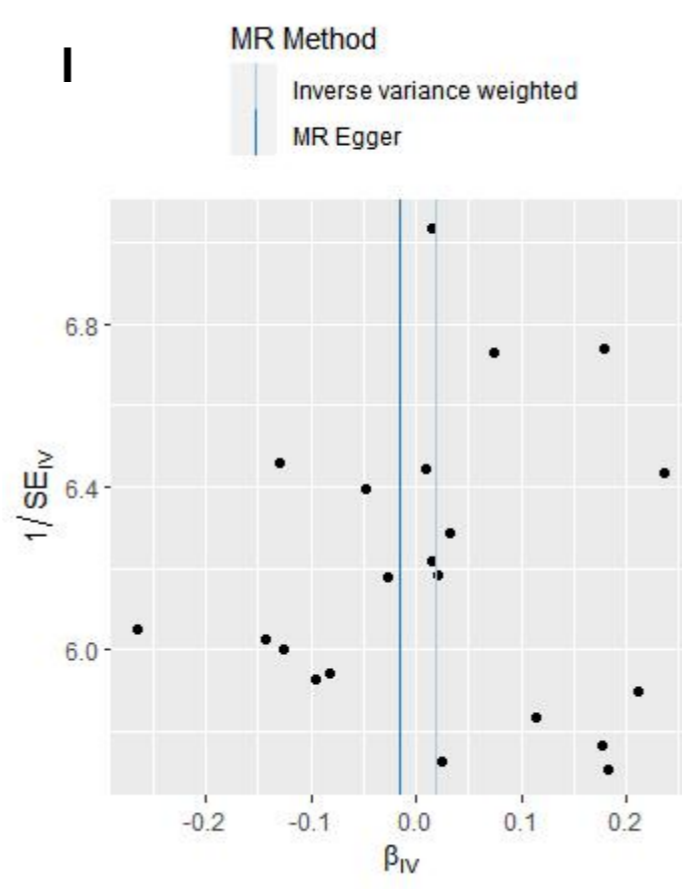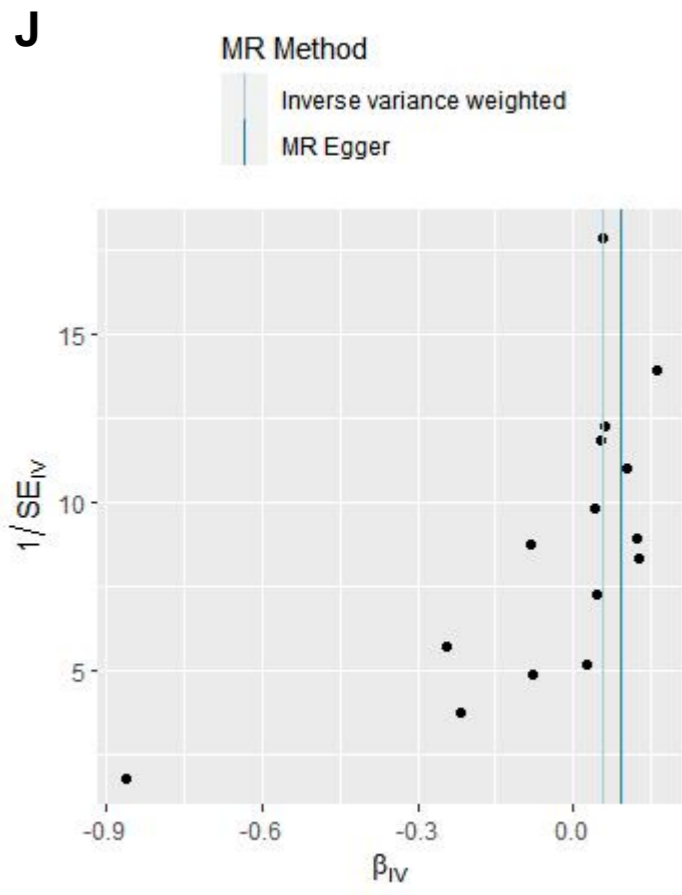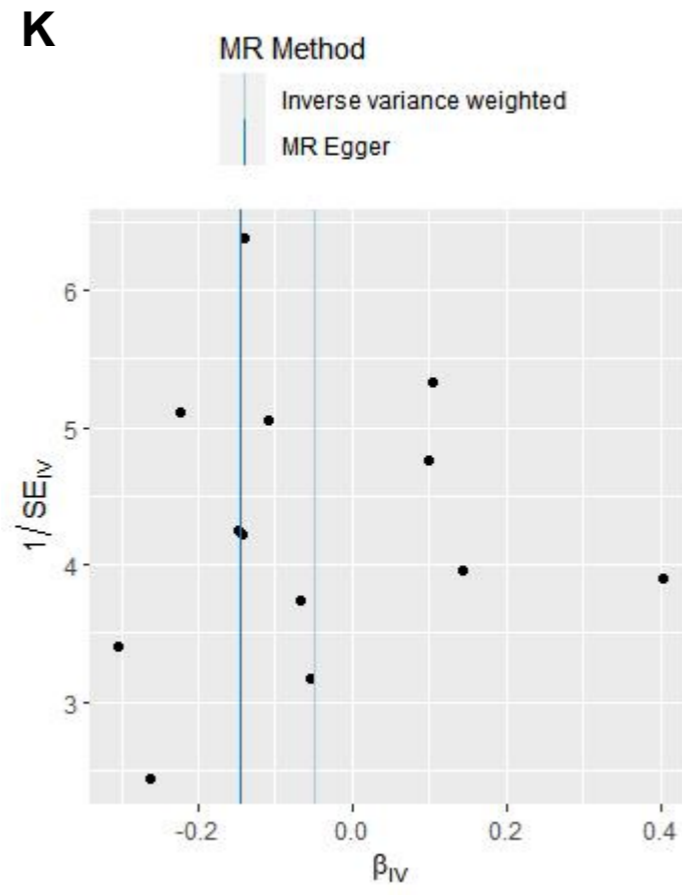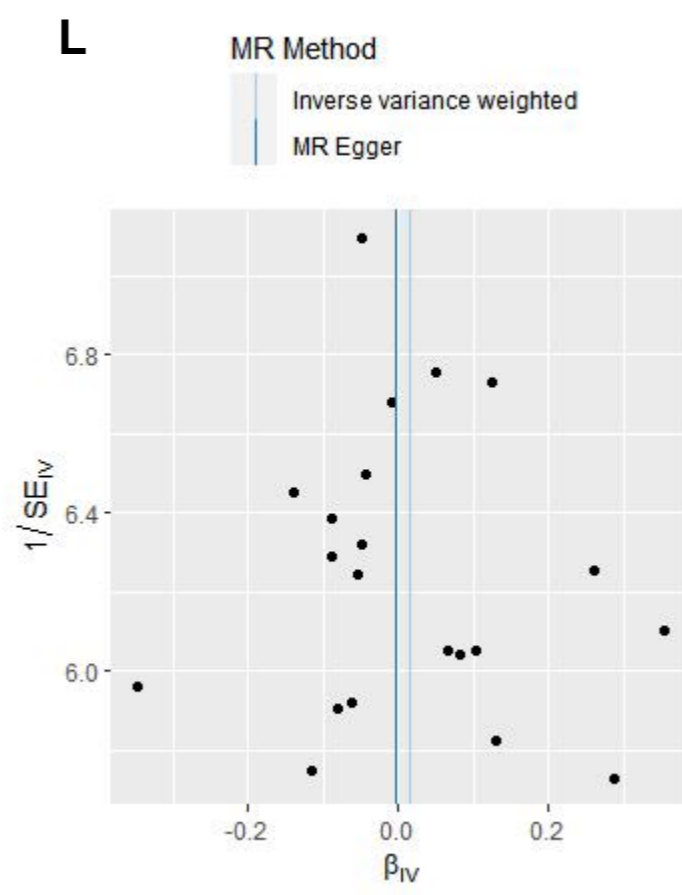

Supplement: Supplementary 3 — Figure 1: funnel plot for IVW and MR-Egger method. [file 2474118.f3.pdf]

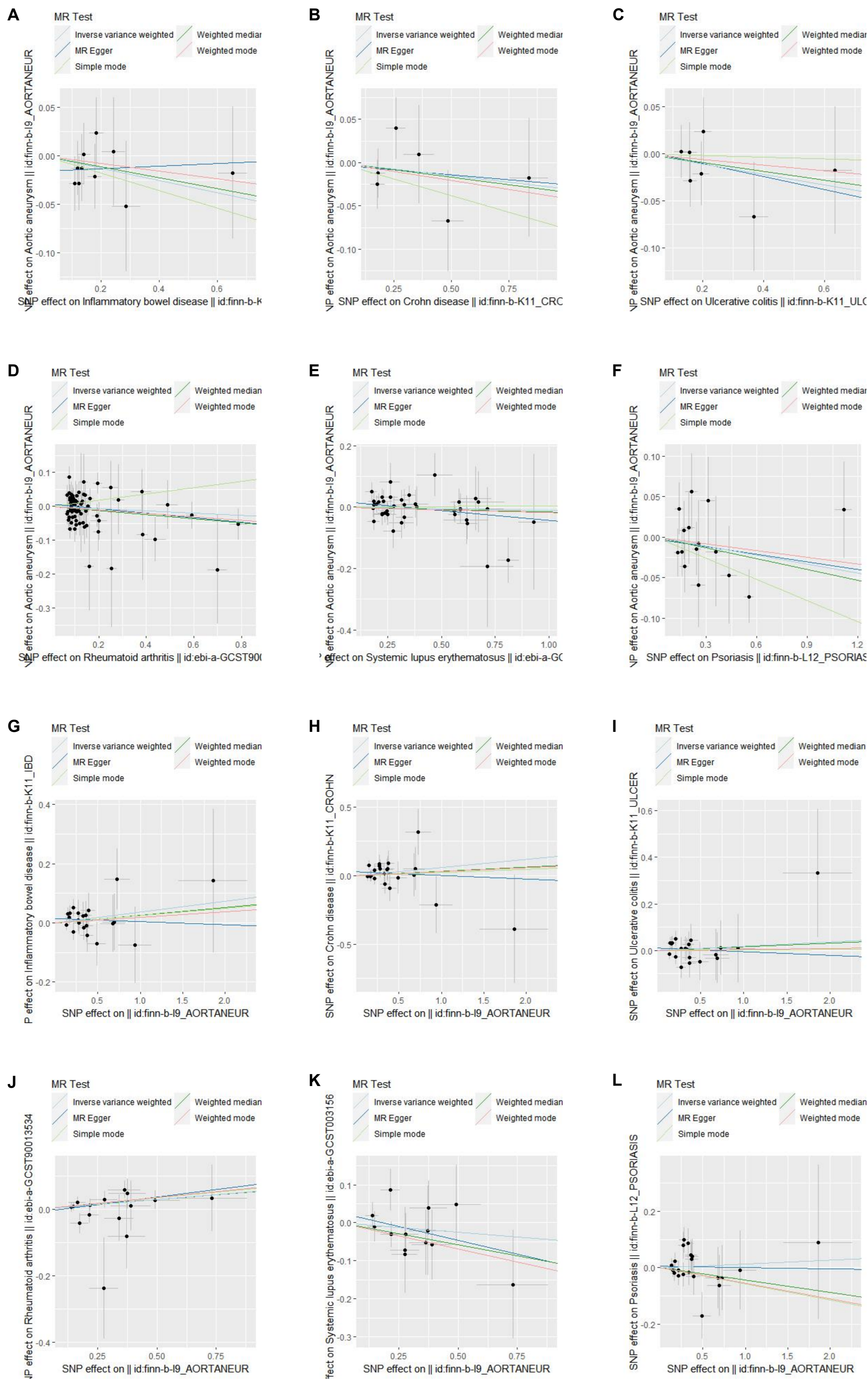

Supplement: Supplementary 6 — Figure 3: scatter plot with all IVs. [file 2474118.f6.pdf]
