# Supplementary material for: The Causal Relationship between Immune-Mediated Inflammatory Diseases and Aortic Aneurysm: A Bidirectional Two-Sample Mendelian Randomization Study
Source: J Immunol Res. 2024 Oct 9;2024:2474118. doi: 10.1155/2024/2474118 (PMC11483648; doi:10.1155/2024/2474118)
Supplement: Supplementary 4 — Figure 2: leave-one-out sensitivity analysis. [file 2474118.f4.pdf]

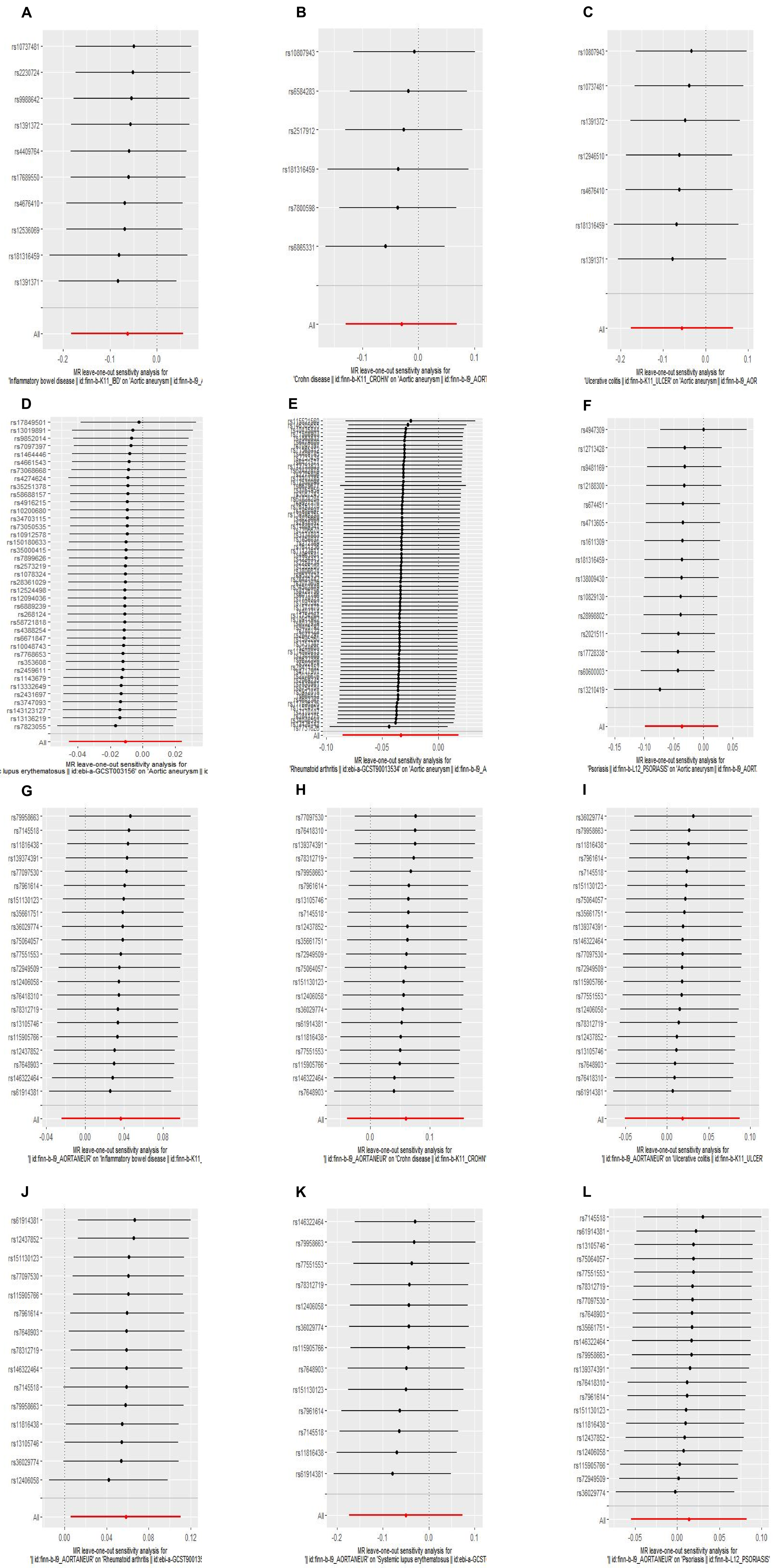

A
B
C
D
E
F
G
H
I
J
K
L
MR leave-one-out sensitivity analysis for
